# Circular RNA network plays a potential antiviral role in the early stage of JEV infection in mouse brain

**DOI:** 10.3389/fmicb.2023.1165378

**Published:** 2024-01-05

**Authors:** Mengli Chen, Lei Kang, Tong Zhang, Jiayang Zheng, Dishi Chen, Donghua Shao, Zongjie Li, Beibei Li, Jianchao Wei, Yafeng Qiu, Xiuli Feng, Zhiyong Ma, Ke Liu

**Affiliations:** ^1^Shanghai Veterinary Research Institute, Chinese Academy of Agricultural Science, Shanghai, China; ^2^Key Laboratory of Animal Disease Diagnostic and Immunology, Department of Veterinary Medicine College, Nanjing Agricultural University, Nanjing, Jiangsu, China; ^3^Sichuan Animal Disease Prevention and Control Center, Chengdu, China

**Keywords:** circular RNA, Japanese encephalitis, Japanese encephalitis virus, ceRNA, miR-709

## Abstract

Japanese encephalitis is one of the most important insect-borne infectious disease with public health concern. The virus can break the blood–brain barrier and cause death or long-term sequela in infected humans or animals. Viral encephalitis is an important clinical feature of JEV infection. In recent studies, CircRNAs and related ceRNAs data illustrated the regulative role in many aspects of biological process and disease duration. It is believed that CircRNA regulates JEV infection in a ceRNA-dependent mechanism. In this study, brain tissues of experimental mice were sequenced and analysised. 61 differentially expressed circRNAs, 172 differentially expressed miRNAs and 706 differentially expressed mRNAs were identified by RNA-Sequencing and statistical analysis. CX3CR1 was determined as a key host factor impact JEV infection by microRNA interference measurement. CX3CR1 interaction network indicated circStrbp/miR709/CX3CR1 as a functional regulation axis. Further sequencing in BV2 cell shown CX3CR1 is a special target of miR-709 only during JEV infection. In summary, our study presented a new ceRNA pathway that impact JEV infection *in vivo* and *in vitro*, which could be a therapeutic target to fight against JEV.

## 1 Introduction

Japanese encephalitis is a zoonotic infectious disease transmitted by mosquitoes, and the pathogen, Japanese encephalitis virus (JEV), belong to *Flaviviridae* which is an important part of arboviruses (Huang et al., 2014). JEV infection can cause viral encephalitis in humans and nervous system or organ infection in animals (Daep et al., 2014). Related to mosquito-borne vectors, Japanese encephalitis mainly spreads in tropical and subtropical regions. Cases of Japanese encephalitis in China mainly occur in southern regions (Zheng et al., 2012; Le Flohic et al., 2013). JEV infection can cause fatal viral encephalitis or viral encephalitis with sequelae in humans, orchitis in boars and abortion in sows, and different diseases in birds (Di et al., 2020; Hameed et al., 2021; Bharucha et al., 2022; Pichl et al., 2022). Therefore, Japanese encephalitis is an infectious disease that endangers public health (Daep et al., 2014).

The greatest threat of JEV infection is the destruction of the host’s brain tissue. Once the virus invades the brain tissue, it will cause an uncontrollable inflammatory response in a short period of time, leading to host fatality (Yun and Lee, 2014). Even if the host survives, the risk of sequelae is 50%. From the perspective of cytokines, this process mainly involves TNF-α, interleukins, and chemokines (Liu et al., 2018). Among them, inflammatory chemokines and homeostasis-regulating chemokines play important roles. We found in previous studies that CCR5- and CCR2-related chemokines (CCL3, CCL4, and CCL5) play key roles in Japanese encephalitis and that these chemokines are potential targets for the treatment of viral encephalitis (Liu et al., 2018). We also found that the brain- and liver-specific chemokine CX3CL1 was downregulated during JEV infection. CX3CL1 is a bifunctional chemokine that participates in the regulation of inflammation and tissue homeostasis. No study has clarified the relationship between the downregulated expression of CX3CL1-CX3CR1 axis and JEV infection of brain. In this study, we explored this issue from the perspective of circRNA-miRNA-mRNA.

In 2013, a large number of circRNAs were discovered with the help of high-throughput sequencing technology, proving that circRNAs are important RNA components in organisms (Memczak et al., 2013). With advancements in research, circRNAs have been proven to act as miRNA sponges and protein sponges and affecting protein expression (Chen, 2016). circRNAs, which act as miRNA sponges, adsorb miRNAs and further regulate the expression of mRNAs, which is the most studied function of circRNAs (Hansen et al., 2013). circRNA-miRNA-mRNA has been proven to play an important role in chronic diseases such as cancer, brain tissue disease, and vascular disease (Wang et al., 2021; Zhu et al., 2021; Xie et al., 2022). The abnormal expression of circRNA slowly promotes changes in miRNA–mRNA expression and gradually leads to the occurrence of chronic diseases. Among the many studies on circRNA-miRNA-mRNA, only a few have focused on circRNA-miRNA-mRNA related to viral infection (Lu et al., 2020; Hu et al., 2021; Li et al., 2021; Shi et al., 2021; Zhao et al., 2021; Chen et al., 2022), in particular acute viral infection, probably due to the weak regulatory effect of circRNA-miRNA-mRNA on viruses. However, a JEV infection model has been shown to be useful for research on viral circRNA-miRNA-mRNA. JEV infection of mouse brain tissue can cause significant changes in circRNA_0000220 and the downstream targets of circRNA_0000220, i.e., miR-326-3p and BCL3/MK2/TRIM25, ultimately affecting the occurrence of viral encephalitis (Li et al., 2020).

In this study, we focused on the early stages of JEV infection of mouse brain tissue and mouse microglia cell. Through high-throughput sequencing, we discovered the network through which circRNA-miRNA regulates CX3CR1 and demonstrated the inhibitory effect of this regulation on JEV. The findings indicate that the changes in circRNA-miRNA in the early stage of infection can regulate the expression of CX3CR1 and affect the early stage of virus infection.

## 2 Materials and methods

### 2.1 Ethics statement

Mouse experiments were performed in compliance with the Guidelines on the Human Treatment of Laboratory Animals (Ministry of Science and Technology of the People’s Republic of China, Policy No. 2006 398) and were approved by the Institutional Animal Care and Use Committee at the Shanghai Veterinary Research Institute (IACUC No: Shvri-Pi-0124).

### 2.2 Cells, viruses and mice

Mouse microglia (BV2) cells, Baby hamster kidney (BHK-21) cells and African green monkey kidney (Vero) cells were purchased from the ATCC (Rockville, Maryland) and maintained in Dulbecco’s modified Eagle’s medium (DMEM) supplemented with 10% fetal bovine serum (FBS) at 37°C in a 5% CO2 incubator.

JEV NJ2008 strain (Genbank No. GQ918133) was used in study. Virus was propagated in BHK-21 cells and titrated in Vero cells.

8 weeks specific pathogen free (SPF) female C57BL/6 mice were grouped and infected in this study. Mice were purchased from Shanghai SLAC Laboratory Animal Co., Ltd. and maintained under pathogen-free conditions.

### 2.3 Mouse infection and sampling

Grouped C57BL/6 mice were injected intraperitoneally (i.p.) with 4 × 10^6^ PFU of JEV or phosphate buffered saline (PBS). All infected or mock infected mice were maintained under pathogen-free conditions in animal central of Shanghai Veterinary Research Institute. The mice were sacrificed at 5, 7, 8, 9 and 10 days after injection, then mouse brain, blood, lymph nodes were collected. Brain samples collected at 7 day post injection were send for CircRNA-miRNA-mRNA sequencing. Other samples for qPCR detection were stored at minus 80°C.

### 2.4 RNA sequencing analysis

Three biological replicates of the infected and mock infected samples at 7 day after injection were used for circRNA, miRNA and mRNA sequencing. For circRNA sequencing, total RNA was extracted and digested with RNase R to degest linear RNA and was further purified by using RNeasy MinElute Cleanup Kit (Qiagen). NEBNext Ultra Directional RNA Library Prep Kit for Illumina was used for constructing the strand-specific library according to the manufacturer’s instructions. The miRNA sequencing libraries were generated by using the Small RNA Sample Pre Kit (Illumina) and the mRNA were enriched by using Oligo(dT) magnetic beads. The next step was similar with circRNAs sequencing. To get the clean data (clean reads), reads containing poly-N or adapter sequence and reads of low-quality were removed. Candidate circRNAs were subjected to blast in the circBase database for annotation, and unannotated circRNAs were recognized as novel ones. miRNAs and mRNAs were aligned by using bowtie tools or to the mouse reference genome (GRCm38/mm10), respectively. The DESeq2 package and the edgeR package (http://www.r-project.org/, accessed 21 May 2019) were applied to analyze differentially expressed circRNAs and mRNAs, respectively. Candidates were considered significantly differentially expressed ones if a fold change 2 and a *p* value <0.05.

In addition to combined sequencing, BV2 cells transfected with siRNA were subjected to RNA-Seq sequencing. Three biological replicates of the infected and mock infected samples were collected, treated and analysed as above.

### 2.5 Quantitative PCR

For circRNA, miRNA, and mRNA expression analysis, total RNA was extracted from mice brain using TRIzol Reagent (Life Technologies). cDNA was immediately reverse-transcribed using RT Master Mix (TaKaRa, Tokyo, Japan). JEV or gene mRNA levels were detected by a SYBR quantitative PCR (qPCR). PCR was performed using an ABI Prism 7,900 sequence-detection system (Applied Biosystems, Foster City, CA, United States), using SYBR Green PCR Master Mix. The amount of target gene expression was calculated from the respective standard curves and normalized using glyceraldehyde-3-phosphate dehydrogenase (GAPDH), and displayed as fold change. Briefly, mock infected mice collected at the same time points were measured and set as 1. Then, JEV infected mice were calculated referenced to mock and presented as fold change. Relative gene expression to the control was determined by the standard 2^−ΔΔCt^ method. Mice chemokine primer was a kindly gift from Dr. Clive S. McKimmie and Dr. Gerard J. Graham.

### 2.6 RNA interference

Small interfering RNAs (siRNAs) targeting the indicated genes were synthesized chemically. A negative siRNA without known target genes was synthesized as control. The siRNA sequences are shown in S1 Table. BV2 cells were plated in DMEM medium containing 10% FBS at 37°C overnight, washed with PBS, and transfected with siRNA at a concentration of 20 nM using Lipofectamine 2000 (Thermo Fisher Scientific), according to the manufacturer’s protocol. The transfectants were re-cultured at 37°C for 24 h and subsequently inoculated with JEV at a MOI of 0.1. JEV replication in the transfectants was determined by qPCR at 24 h post-infection (hpi).

### 2.7 Statistical analysis

All the measurements were conducted in triplicate in at least three independent experiments. Mean values ± standard deviation (SD) was calculated using Microsoft Excel. Statistical analysis was done by Student’s t test or log rank test and values were considered significant when *p* < 0.05. Figures were made using the GraphPad™ Prism 5.0 software.

## 3 Results

### 3.1 Expression characteristics of circRNAs in JEV-infected mouse brain tissue

**Figure 1:**
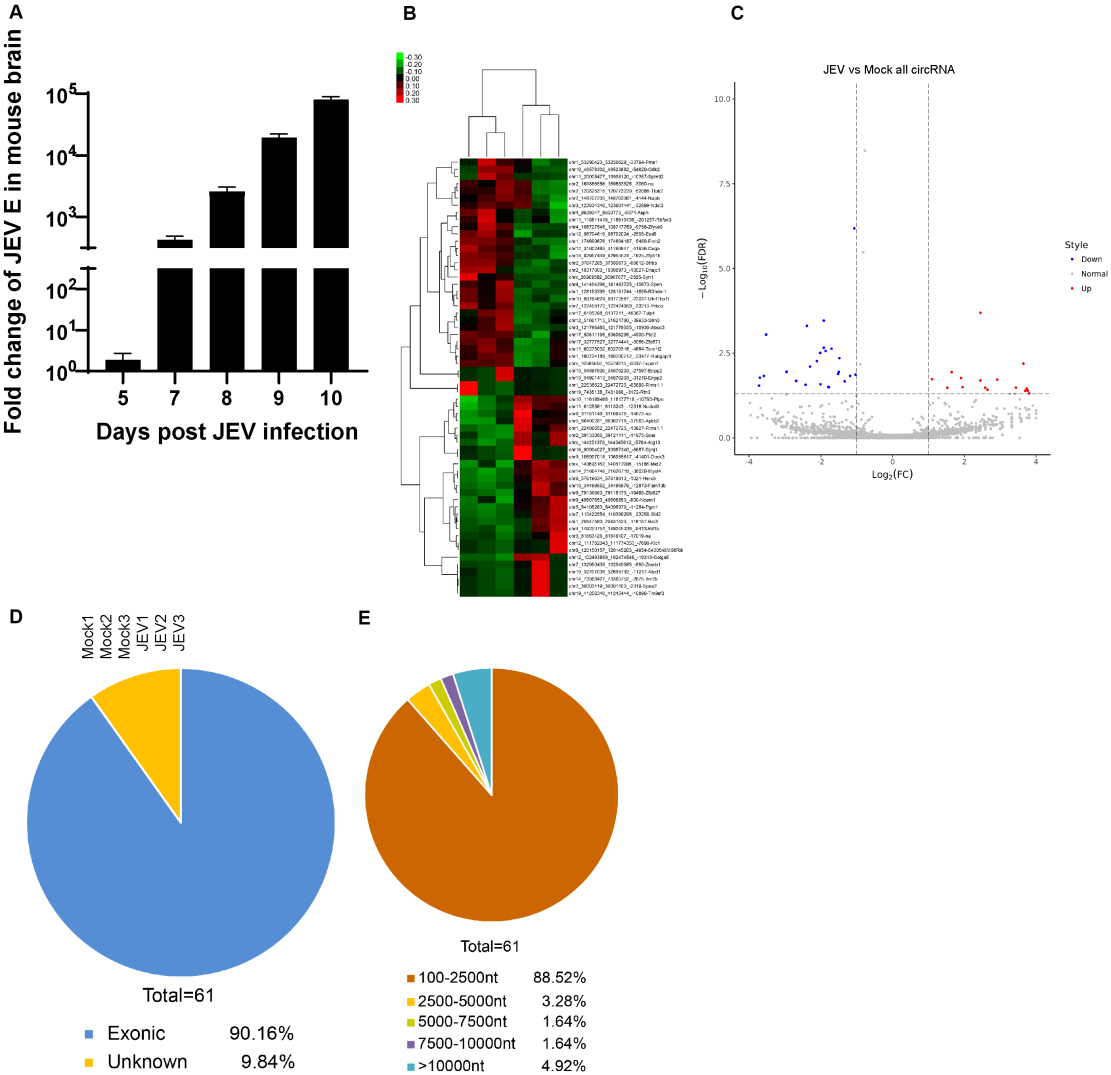
The characteristics of circRNA during JEV infection in mouse brain. **(A)** Viral fold change in mouse brain. JEV (4 × 10^6^ PFU) was injected intraperitoneally. The viral load was determined by detecting JEV envelope **(E)** gene and normalized with mouse GAPDH. All fold changes were measured by Real-time PCR. Data are shown as the mean ± standard error (SEM). Six mice for each group were detected, and the experiments were repeated three times. **(B,C)** Heat map **(B)** and Volcanic map **(C)** of differently expressed circRNAs enriched in mouse brain. Red color represents up-regulations and other colors represent down-regulatons. Difference were calculated as significant if a fold change >2 and a *p* value <0.05. **(D)** The genomic distribution of differently expressed circRNAs. Exonic, intron and unknown distribution were measured. **(E)** The length distribution of differently expressed circRNAs. Length range from 100 to more than 10,000 were analysed.

To analyze the characteristics of circRNA expression changes in JEV-infected mouse brain tissue in the early stage, JEV was intraperitoneally injected into mice, and brain tissue samples were collected 7 days after infection (Figure 1A). The samples were processed for high-throughput sequencing analysis.

First, the differential expression of circRNAs in JEV-infected mouse brain tissue was analyzed. Through second-generation high-throughput sequencing, a total of 29,129 circRNAs were detected in mouse brain tissue samples (Supplementary Figure S1A). A total of 74.82% of these circRNAs were 100 to 2,500 nt long, and 15.86% of these circRNAs were longer than 10,000 nt (Supplementary Figure S1B). Therefore, most of the identified circRNAs fell within these 2 length ranges. Chromosomes derived from circRNAs were evenly distributed, and the chromosomal origins of circRNAs were not significantly different between the JEV-infected group and the uninfected group (mock group), both with chr2 as the main origin (Supplementary Figure S1C).

The differential expression of total circRNAs between the JEV-infected and mock groups was analyzed (2-fold change in expression, false discovery rate (FDR) < 0.05), and 61 circRNAs were found to be differentially expressed between the 2 groups (Figure 1B). In a comparison with the circRNAs in the mock group, there were 33 downregulated circRNAs and 28 upregulated circRNAs in the JEV-infected group (Figure 1C). A total of 90.16% of the 61 differentially expressed circRNAs were derived from exons (Figure 1D), and 88.52% of the 61 differentially expressed circRNAs were 100 to 2,500 nt long (Figure 1E). The small portion (61/29,129) of differentially expressed circRNAs after JEV infection indicates that the circRNAs were generally little affected by JEV infection. This result is consistent with the results of the previously published article (Li et al., 2020).

### 3.2 Cerna network in the brain tissue of JEV-infected mice

**Figure 2:**
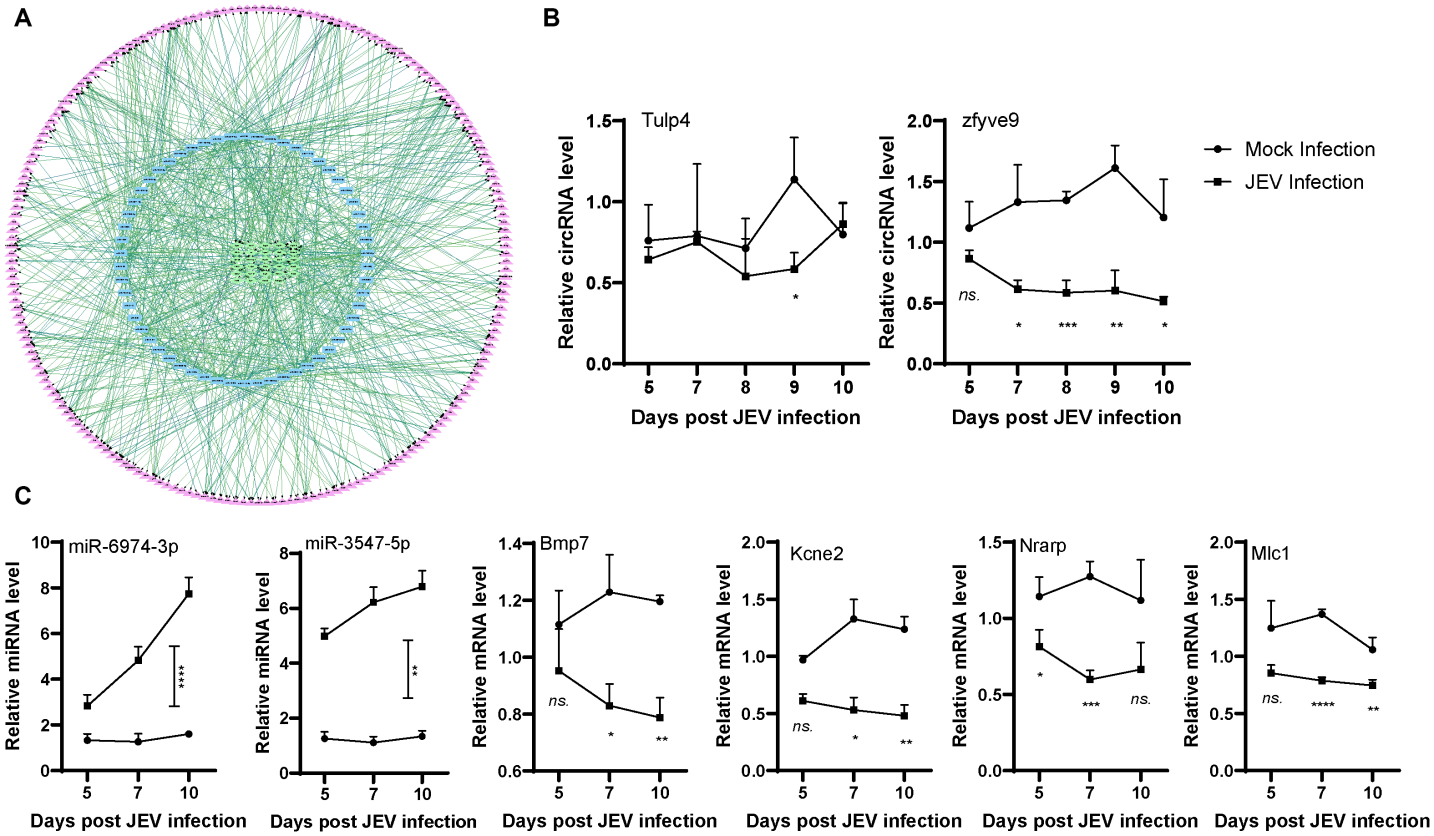
Network of differently expressed ceRNA. **(A)**. circRNA (green)-miRNA (blue)-mRNA (purple) interaction network. CircRNA is inside the circle with green frame, miRNA is in the middle of the circle with blue frame, mRNA is outside the circle with purple frame. **(B,C)**. Validation of circRNAs, miRNAs and mRNAs expression in infected mouse. Mice were infected or mock infected with JEV. Mouse brain was sampled in the indicated time points, then representative circRNAs, miRNAs and mRNAs were detected by qPCR.

In addition to circRNAs, high-throughput sequencing of miRNAs and mRNAs in mouse brain tissue was also performed. A total of 172 differentially expressed (fold change>2, *p < 0.05*) miRNAs (Supplementary Figures S2A,B) and 706 differentially expressed mRNAs (Supplementary Figures S2C,D) were obtained from the analysis of total miRNAs and mRNAs. Then, the competing endogenous RNA (ceRNA) network was obtained from a correlation analysis of circRNAs, miRNAs and mRNAs. In the circRNA-miRNA-mRNA interaction network (Figure 2A), circRNAs act as adsorption sponges with mRNAs as the ultimate targets, and miRNAs bridge circRNAs and mRNAs and act as mediators that directly interact with circRNAs and mRNAs (Hansen et al., 2013). Therefore, we organized a structure of the ceRNA interaction network centered on miRNA. There were 136 miRNAs in the ceRNA network, most of which were upregulated, to a maximum fold change of 20. Each miRNA generally adsorbed 1–11 circRNAs, but most miRNAs adsorbed 2–5 circRNAs. Each miRNA generally targeted 1–42 mRNAs, but most miRNAs targeted approximately 10 mRNAs (Supplement files). The number of genes adsorbed and targeted and the binding efficiency of miRNAs are key factors that affect the effectiveness of miRNAs. Using circMir software, the free energy of an miRNA binding with circRNAs and mRNAs was analyzed to estimate the binding efficiency of the miRNA. miRNA background expression level and fold change are other factors that affect the effectiveness of miRNAs. Specifically, the higher the expression of a functional miRNA, the stronger is the potential biological effect of the miRNA. The binding targets of each miRNA were scored based on the targets, free energy, and expression levels in the ceRNA data. A ceRNA interaction network diagram was obtained using the ceRNA interaction network and these scores (Table 1).

**Table 1:** Profile of ceRNA in JEV infected mouse brain based on miRNA.

miRNA	Log2FC	FDR	Style	Number of miRNA-mRNA	Number of miRNA-CircRNA
mmu-miR-1198-3p	4.354	0.000049599122204674	Up	5	1
mmu-miR-1224-3p	6.318	0.0351095494688527	Up	24	3
mmu-miR-136-5p	−1.822	0.0487122507988076	down	1	4
mmu-miR-141-3p	3.212	0.0174460832523778	up	4	4
mmu-miR-142a-3p	2.780	0.0362638626102003	up	2	2
mmu-miR-149-3p	4.232	0.000319079971256809	up	35	2
mmu-miR-155-5p	4.285	0.000634899620817067	up	2	2
mmu-miR-1668	5.258	0.0251796787996459	up	6	3
mmu-miR-1892	7.261	0.0407966651019063	up	24	5
mmu-miR-1893	7.007	0.00899413636337472	up	1	1
mmu-miR-1897-5p	4.832	0.000342747015051776	up	4	3
mmu-miR-1903	4.029	0.00384701087453602	up	11	11
mmu-miR-1904	3.837	0.00331039855704739	up	16	7
mmu-miR-1906	5.117	0.00412344272229418	up	30	5
mmu-miR-191-5p	−1.861	0.04255097434091	down	1	1
mmu-miR-1934-3p	5.337	1.67489304823256E-07	up	4	3
mmu-miR-1934-5p	5.071	0.0000669276227204325	up	11	6
mmu-miR-1943-5p	4.051	0.00234750264197207	up	32	3
mmu-miR-1946a	5.411	8.93702845873959E-06	up	7	3
mmu-miR-1946b	5.228	0.0000193173326343373	up	7	1
mmu-miR-1954	4.545	0.00141347398704677	up	15	3
mmu-miR-1957a	5.641	2.80951635748803E-10	up	4	1
mmu-miR-1957b	4.206	0.000431442637061225	up	5	1
mmu-miR-1967	6.135	0.000997225904950666	up	8	2
mmu-miR-199b-5p	−1.901	0.0340954165590419	down	13	6
mmu-miR-200c-5p	5.198	0.00141347398704677	up	1	2
mmu-miR-219a-1-3p	4.980	0.00625810142621969	up	3	2
mmu-miR-21a-3p	2.172	0.0494699059120279	up	12	4
mmu-miR-2861	7.318	0.00385392970056958	up	13	4
mmu-miR-3061-5p	4.403	0.00163651041239826	up	10	1
mmu-miR-3112-3p	4.299	0.0277535892236198	up	2	3
mmu-miR-320-5p	5.102	0.0259077898099045	up	21	7
mmu-miR-326-3p	−2.988	0.000103988200795178	down	21	9
mmu-miR-329-5p	−1.927	0.0273670106681081	down	7	4
mmu-miR-346-3p	6.484	2.46384407557472E-11	up	34	3
mmu-miR-3470a	5.605	2.35273705619275E-09	up	9	5
mmu-miR-3470b	5.850	2.40270286547131E-11	up	11	2
mmu-miR-3473a	5.422	0.0000400854861074338	up	8	2
mmu-miR-3473b	6.386	2.46384407557472E-11	up	18	4
mmu-miR-3473c	5.956	4.97147489813369E-11	up	19	5
mmu-miR-3473e	6.984	1.06955113712167E-12	up	18	3
mmu-miR-3547-5p	6.587	0.00036619368479388	up	37	9
mmu-miR-3620-5p	5.693	0.00287623388474358	up	18	5
mmu-miR-376a-3p	−1.864	0.0425997704711526	down	1	1
mmu-miR-3960	7.308	0.00141347398704677	up	5	1
mmu-miR-455-3p	−2.341	0.00891332667445275	down	14	6
mmu-miR-466i-5p	4.276	0.0000334778362810868	up	28	1
mmu-miR-466j	3.803	0.0166799939343183	up	20	3
mmu-miR-466 k	4.700	7.96549854883085E-06	up	29	3
mmu-miR-495-3p	−1.910	0.0314062492584068	down	4	2
mmu-miR-5098	4.775	0.000174242767966446	up	1	1
mmu-miR-5106	8.347	1.08005791442604E-12	up	16	4
mmu-miR-5112	4.523	0.0000530957128344883	up	11	3
mmu-miR-5114	4.496	0.0169258868672246	up	15	5
mmu-miR-5121	6.117	0.00141347398704677	up	4	2
mmu-miR-5128	6.121	0.0020577634593746	up	5	1
mmu-miR-5130	6.368	0.0173183661799466	up	9	1
mmu-miR-532-5p	−2.423	0.0088738742848416	down	11	5
mmu-miR-543-3p	−1.859	0.0487122507988076	down	1	3
mmu-miR-615-5p	6.624	0.00141347398704677	up	6	2
mmu-miR-6238	4.213	0.000174548270725916	up	1	1
mmu-miR-6366	5.179	0.0127278535486286	up	12	1
mmu-miR-6368	3.974	0.0234283989841492	up	25	7
mmu-miR-6372	5.018	0.0407966651019063	up	6	6
mmu-miR-6380	4.625	0.0159604685145588	up	4	2
mmu-miR-6393	8.166	0.00385392970056958	up	9	2
mmu-miR-6404	6.210	0.0296114257249124	up	19	7
mmu-miR-652-5p	−2.241	0.0373874212508746	down	8	1
mmu-miR-665-3p	−1.961	0.0325912135152282	down	33	8
mmu-miR-677-3p	5.031	0.0032312965958258	up	22	5
mmu-miR-680	7.085	0.00504177392794773	up	10	5
mmu-miR-683	2.568	0.0456928790631196	up	7	7
mmu-miR-690	6.103	2.29537891480982E-09	up	2	2
mmu-miR-692	6.005	0.00190160169504928	up	4	5
mmu-miR-6921-5p	6.131	0.0407966651019063	up	16	4
mmu-miR-6925-5p	6.151	0.02019673764755	up	26	3
mmu-miR-6931-5p	6.986	0.000297048033031346	up	42	3
mmu-miR-6936-5p	5.273	0.0174460832523778	up	11	3
mmu-miR-6937-5p	4.823	0.0000418802801207815	up	10	3
mmu-miR-6955-3p	5.719	0.00759222820514879	up	8	3
mmu-miR-6957-5p	8.234	7.32101541376813E-10	up	14	4
mmu-miR-696	10.141	2.18807307533137E-10	up	4	1
mmu-miR-6961-5p	20	0.00839672354830158	up	8	4
mmu-miR-6963-3p	4.086	0.0374168535124453	up	11	4
mmu-miR-6963-5p	5.294	0.0484222299598572	up	18	5
mmu-miR-6971-5p	5.890	0.0251085195447356	up	27	2
mmu-miR-6973a-5p	12.804	4.0347785792735E-07	up	9	1
mmu-miR-6973b-5p	6.025	0.0243289483982281	up	10	1
mmu-miR-6974-3p	6.904	0.0313736349917696	up	19	6
mmu-miR-6978-5p	5.493	0.000742500462525439	up	8	1
mmu-miR-6979-5p	4.473	0.0251085195447356	up	14	1
mmu-miR-6981-3p	20	0.0238138902402119	up	11	2
mmu-miR-6981-5p	5.266	0.000375612841723136	up	34	3
mmu-miR-6989-5p	5.119	0.0494699059120279	up	13	3
mmu-miR-6992-5p	4.582	0.0243289483982281	up	16	9
mmu-miR-6995-3p	4.504	0.000963078614648598	up	10	4
mmu-miR-7007-5p	4.943	0.00442283369163749	up	24	3
mmu-miR-7011-3p	5.346	0.0000814711849479471	up	13	2
mmu-miR-7011-5p	4.755	0.00418383682182131	up	26	1
mmu-miR-7014-5p	4.709	0.0127123630058442	up	15	3
mmu-miR-7017-3p	6.107	0.0251153852432307	up	12	2
mmu-miR-7023-5p	6.398	0.0427202026166347	up	23	2
mmu-miR-7025-5p	4.678	0.014602157096298	up	18	1
mmu-miR-7028-3p	9.172	0.0205384032785827	up	32	7
mmu-miR-7032-5p	5.301	0.00134000605927864	up	21	4
mmu-miR-7033-5p	6.178	4.98773663785387E-06	up	38	10
mmu-miR-7042-3p	20	0.0422811649114925	up	10	3
mmu-miR-7044-5p	4.936	0.0374168535124453	up	14	3
mmu-miR-7045-5p	5.464	0.0242939518092793	up	7	1
mmu-miR-7051-3p	5.078	0.000584840384232765	up	17	6
mmu-miR-7068-5p	4.369	0.0137436651698999	up	13	2
mmu-miR-7070-5p	4.943	0.00571628977371187	up	19	4
mmu-miR-7075-5p	7.202	0.00322989034525318	up	27	3
mmu-miR-7082-5p	5.284	0.00812302360336593	up	8	2
mmu-miR-7084-3p	7.307	0.0000970351783144778	up	10	5
mmu-miR-709	7.799	1.8644972480033E-16	up	28	5
mmu-miR-7118-5p	6.996	0.0316024941145538	up	39	4
mmu-miR-712-3p	7.126	1.13403265924996E-13	up	2	2
mmu-miR-712-5p	7.589	1.06955113712167E-12	up	9	5
mmu-miR-7212-5p	7.727	0.0000772767854116384	up	20	2
mmu-miR-7213-3p	6.582	1.09497097213404E-12	up	3	2
mmu-miR-7216-5p	4.638	0.0315857216967794	up	29	3
mmu-miR-7225-3p	5.331	0.0407966651019063	up	3	2
mmu-miR-761	4.228	0.0211170875064135	up	13	2
mmu-miR-762	7.740	5.35534475331443E-16	up	36	7
mmu-miR-7654-3p	5.229	0.0250692218211853	up	1	2
mmu-miR-7654-5p	7.801	0.000282589868205336	up	1	2
mmu-miR-7669-3p	3.834	0.0029078687003109	up	24	3
mmu-miR-8097	6.564	0.0127278535486286	up	4	1
mmu-miR-8102	4.973	0.00516535079654147	up	2	1
mmu-miR-8104	6.019	0.020721000301402	up	12	4
mmu-miR-8106	20	0.00565663613096242	up	2	3
mmu-miR-8112	3.216	0.00188488077250827	up	3	1
mmu-miR-8115	4.552	0.00152129494770112	up	2	2
mmu-miR-8119	5.095	0.0000811944763272297	up	20	3
mmu-miR-92b-3p	−2.518	0.0020577634593746	down	1	1

For the ceRNA network, the circRNAs (downregulated) Tulp4 and zfyve9, miRNAs (upregulated) miR-6974-3p and miR-3547-5p, and mRNAs (downregulated) Bmp7, Kcne2, Nrarp, and Mlc1 were randomly selected for qPCR validation analysis. High-throughput sequencing samples indicated differential changes at one time point. To further understand these biological processes, we infected mice with the same titer of JEV for validation and collected tissue samples 5–10 days after infection to detect changes in gene expression in a time-dependent manner. The expression levels of Tulp4 and zfyve9 were significantly lower in the brain tissue of infected mice than in the brain tissue of mice in the mock group, and zfyve9 expression was generally stable in the infected mice, showing a slow downward trend over time after infection (Figure 2B). The expression levels of miR-6974-3p and miR-3547-5p were significantly higher in the brain tissue of infected mice than in the brain tissue of mice in the mock group and increased with over time after infection. The expression levels of Bmp7, Kcne2, Nrarp and Mlc1 were significantly lower in the JEV-infected group than in the mock group. Except for Nrarp, the expression levels of the other mRNAs decreased over time after infection (Figure 2C). These findings are mostly consistent with the ceRNA high-throughput sequencing results, indicating that the ceRNA high-throughput sequencing results were reliable and that the changes in expression for each gene in the ceRNA network after JEV infection are time dependent.

### 3.3 The chemokine CX3CR1 is regulated by circRNA-miRNA

The ceRNA network regulates many target genes and exerts complex biological functions. We sought to identify a ceRNA network related to viral encephalitis. The sequencing data indicted that CX3CR1 is affected by a series of miRNAs and circRNAs. To better analyze the central role of miRNAs, we scored individual miRNAs according to expression level, binding free energy and number of target genes. High expression levels and low free energy are important factors for adding scores. In addition, we hypothesized that too many target molecules of miRNA would reduce the biological functional strength of miRNAs, so the number of target molecules is negatively correlated with miRNA scoring (Yang et al., 2021). In our ceRNA network, 9 miRNAs targeted CX3CR1, and these 9 miRNAs were scored based on expression, binding free energy and number of targets comprehensively (Table 2). Among the 9 miRNAs, miR-709 had the highest score, implying that miR-709 is an important miRNA that may affect the expression of CX3CR1. These miRNAs are upregulated (4 to 8-fold) after JEV infection, and each miRNA adsorbed 2 to 9 circRNAs, among which Strbp, Tulp4, and Zfyve9 were the most frequently occurring circRNAs, indicating that these 3 circRNAs are likely to bind miRNAs through adsorption and further regulate the expression of CX3CR1.

**Table 2:** Cx3cr1 related miRNA-circRNA.

miRNA	Log2FC	FDR	Style	miRNA-mRNA	CX3CR1 target score	miRNA-circRNA	CircRNA name
mmu-miR-709	7.799	1.8644972480033E-16	up	28	177	5	na Uhrf1bp1l Strbp Tulp4 Strn3
mmu-miR-3473c	5.956	4.97147489813369E-11	Up	19	171	5	Ttbk2 Rims1.1 Plcl2 Eml5 Strn3
mmu-miR-3547-5p	6.587	0.00036619368479388	up	37	155	9	na Grik2 Zfyve9 Cog5 Zfp516 Rims1.1 Strbp Zfp871 Spen
mmu-miR-6936-5p	5.273	0.0174460832523778	up	11	154	3	Zfyve9 Zfp516 Syn1
mmu-miR-6937-5p	4.823	0.0000418802801207815	up	10	150	3	Uhrf1bp1l Zfp871 Spen
mmu-miR-6963-5p	5.294	0.0484222299598572	up	18	154	5	na Rbfox3 Zfyve9 Strbp Zfp871
mmu-miR-6971-5p	5.890	0.0251085195447356	up	27	152	2	na Zfp516
mmu-miR-6992-5p	4.582	0.0243289483982281	up	16	160	9	na Grik2 Rbfox3 Uhrf1bp1l Zfp516 Strbp Plcl2 Spen Tulp4
mmu-miR-7023-5p	6.398	0.0427202026166347	up	23	166	2	Zfyve9 Zfp516

**Figure 3:**
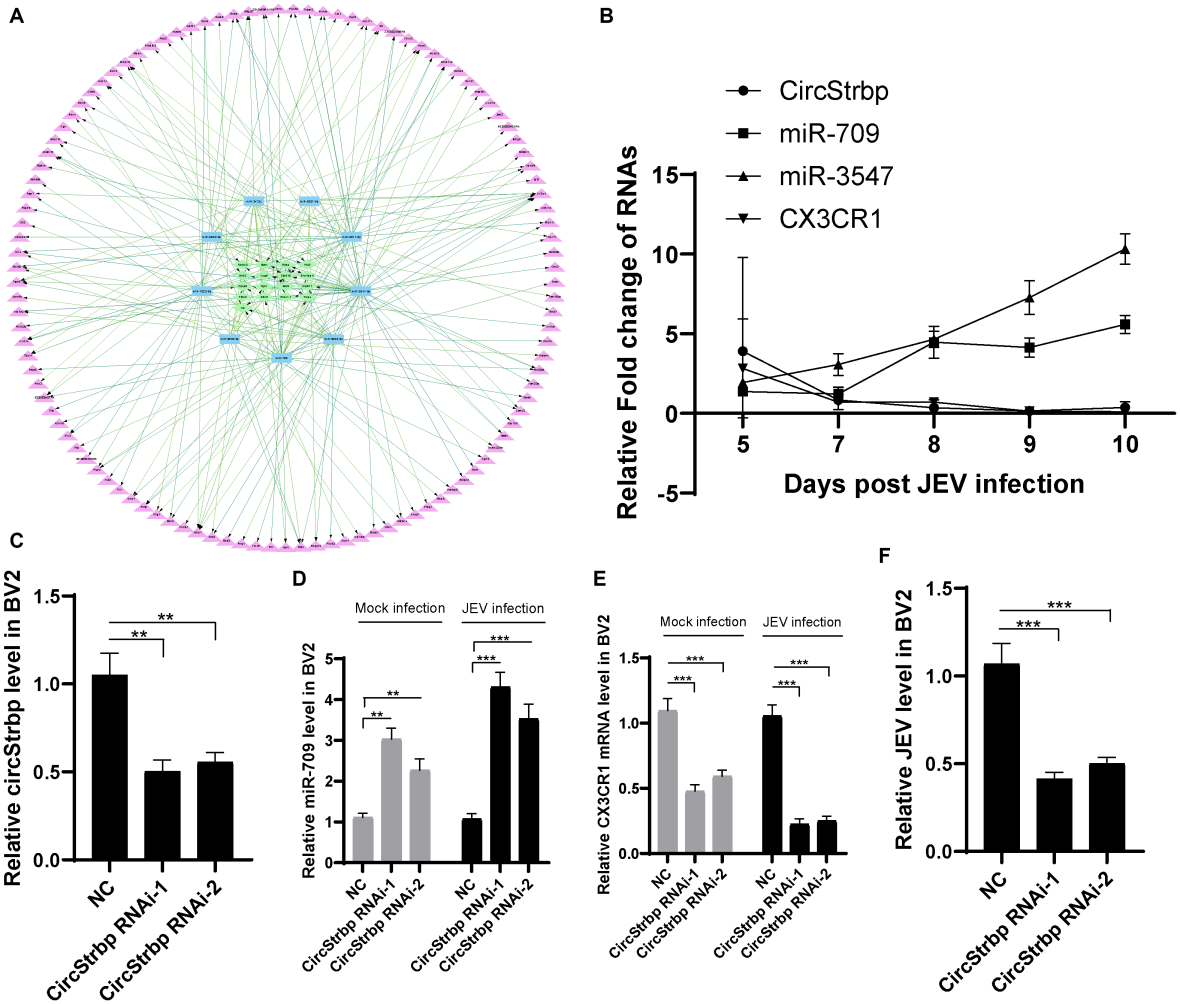
Network of CX3CR1 related ceRNA. **(A)** circRNA (green)-miRNA (blue)-mRNA (purple) interaction network. **(B)** Validation of CX3CR1 related circRNAs, miRNAs and mRNAs expression in infected mouse. Each genes were measured by qPCR using 2^−ΔΔCt^ method and then normalized by a mock sample at day 5 to calculate fold change. **(C)** RNA interference efficiency of circStrbp. CircStrbp gene was measured by qPCR with primers target to linkage sequences of circStrbp. **(D)** Detection of miR-709 levels in BV2 cell after circStrbp knock-down. **(E)** Detection of CX3CR1 mRNA levels in BV2 cell after circStrbp knock-down. **(F)** Detection of JEV mRNA levels in BV2 cell after circStrbp knock-down. All experiments were measured by qPCR using 2^−ΔΔCt^ method and then normalized by a mock. All experiments were repeated three times. Data are shown as means ± standard errors of the means.

The ceRNA network is complex, and miRNAs and circRNAs targeting CX3CR1 also regulate other genes (Figure 3A; Table 2). From another perspective, the network serves to selectively regulates the expression of CX3CR1, further reflecting the complexity of biology. To confirm that CX3CR1 is regulated by circRNA-miRNA, we selected the highest scoring miRNA, miR-709, for *in vitro* validation. First, qPCR was used to analyze the expression of miR-709, circStrbp, and CX3CR1 in JEV-infected mouse brain tissue, with miR-3547 used as a control. The results indicated that the expression level of miR-709 increased over time after infection and that the expression levels of circStrbp and CX3CR1 decreased over time after infection (Figure 3B). The trend of circStrbp-miR-709-CX3CR1 expression detected by qPCR was consistent with the ceRNA results obtained by high-throughput sequencing, indicating a potential regulatory relationship among circStrbp-miR-709-CX3CR1.

To further analysis the ceRNA network function in JEV infection, circStrbp RNAi method was employed. The BV2 cells were transfected with circStrbp siRNA or normal control (NC), then inoculated with 0.1 MOI of JEV 24 h after transfection, and collected for detection 24 h after infection. Interference efficiency of circStrbp siRNA was firstly measured by using qPCR. circStrbp was significantly decreased in siRNA transfeced cells (Figure 3C). As expected, circStrbp related miR-709 and CX3CR1 mRNA were significantly changed after circStrbp RNAi consistent with mouse results (Figures 3D,E). The same results were observed both in the JEV-infected and uninfected groups. Next, JEV mRNA levels were compared in circStrbp RNAi and NC cells. JEV mRNA levels were significantly decreased in circStrbp RNAi cells (Figure 3F). This results implied CX3CR1 enhance JEV infection in BV2 cell which regulated by circStrbp-miR709-CX3CR1 axis.

### 3.4 Mir-709 is an important regulatory miRNA in JEV infection

**Figure 4:**
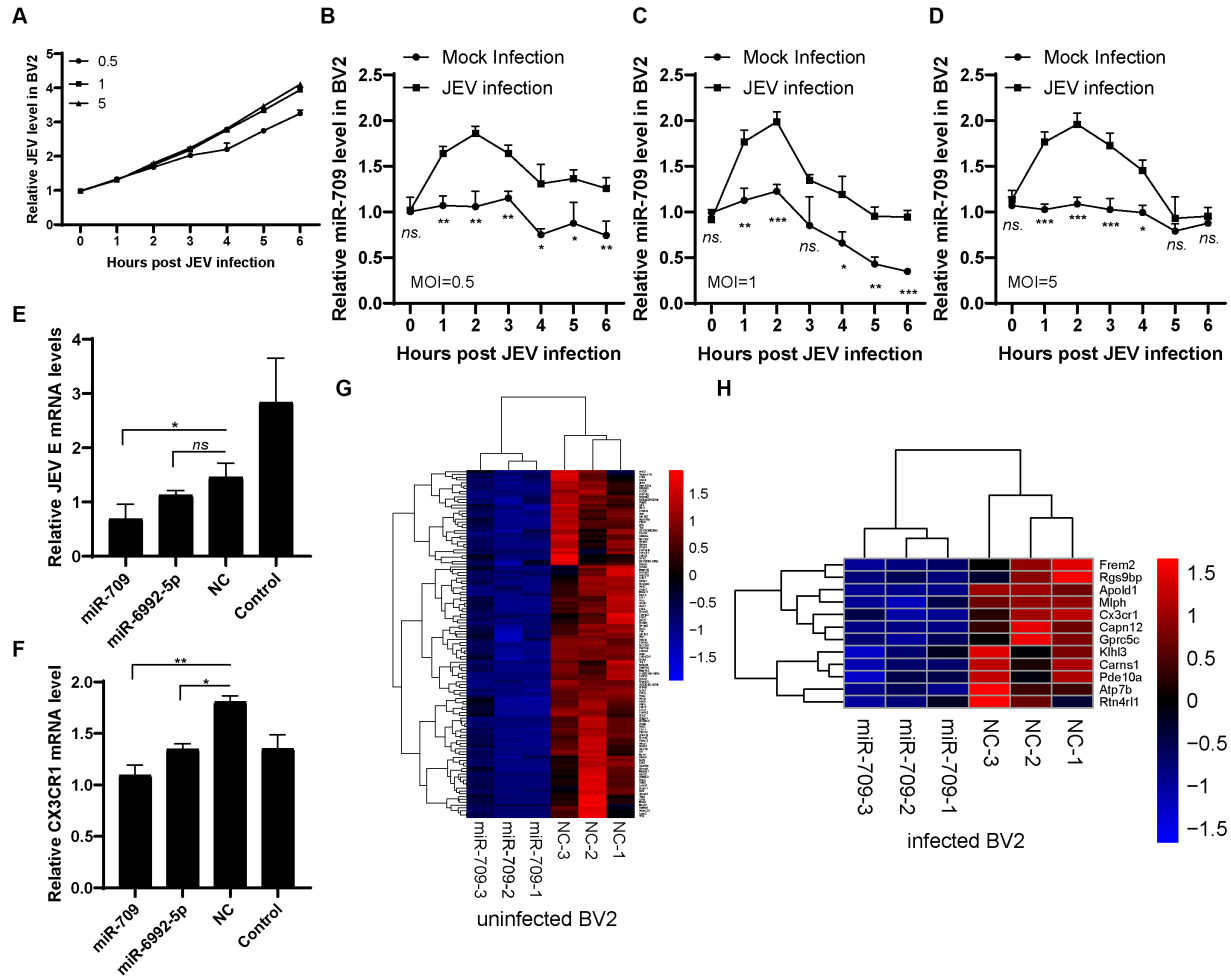
miR-709 targeted CX3CR1 during JEV infection. **(A)** JEV infection curve in BV2 cell. Three dose of JEV (MOI = 0.5, 1, 5) were inculated into BV2 cell, the infected or mock infected cells were collected at different time points. **(B–D)** miR-709 dynamic response after JEV infection in BV2 cell with different MOI. The infected or mock infected cells were collected at different time points, the relative expression of miR-709 was measured by qPCR. Data are shown as the mean ± standard error (SEM). The experiments were repeated three times. **(E)** JEV replication after miRNA transfection. miR-709, miR-6992-5p or NC were transfected in BV2 cell, JEV was inoculated at 24 h after transfection. 24 h post infection, cell samples were collected. **(F)** CX3CR1 mRNA changes after miRNA transfection. miR-709, miR-6992-5p or NC were transfected in BV2 cell, 24 h post transfection, cell samples were collected. *, *p* < 0.05 compared between groups. Data are shown as the mean ± standard error (SE). The experiments were repeated for three times. **(G)** Down-regulation of mRNAs of miR-709 transfected uninfected-BV2 cell. **(H)** Down-regulation of mRNAs of miR-709 transfected infected-BV2 cell. Red color represents up-regulations and blue colors represent down-regulatons. Difference were calculated as significant if a fold change >2 and a *p* value <0.05.

miR-709 has been shown to play a role in regulating JEV infection. As a multitarget miRNA, miR-709 has many potential targets. To further clarify the target molecules of miR-709 and the network of miR-709 in JEV infection, BV2 cells were employed to study miR-709. JEV mRNA levels were measured in 0 to 6 h after JEV infection (MOI = 0.5, 1, 5) (Figure 4A). JEV growth curve was observed in different time points. Therefore, we detected early JEV infection in BV2 cells, consistent with the stage of infection in mice brain. The expression of miR-709 was increased within 1 h of JEV infection, then gradually decreased, and remained relatively low after 6 h (Figures 4B–D). This gradually decreasing may be due to the characteristics of BV2 cells. During the entire infection process, the expression of miR-709 in the JEV-infected group was higher than that in the mock group, and the trend of miR-709 expression in BV2 cells was consistent with that in mouse brain tissue, indicating that BV2 cells can be used as a cell model for studying miR-709. Further validation of the targeting effect of miR-709 on CX3CR1 and JEV was performed using BV2 cells. The BV2 cells were transfected with miR-709 or normal control (NC), inoculated with 0.1 MOI of JEV 24 h after transfection, and collected for detection 24 h after inoculation. The results indicated that miR-709 interfered with the expression of CX3CR1 and that the CX3CR1 mRNA expression was significantly decreased in miR-709-transfected cells (Figure 4F). JEV mRNA expression was lower in CX3CR1 knockdown cells. CX3CR1 promoted the JEV infection of microglia (Figure 4E). The above results demonstrate that miR-709 is regulated by circRNAs and changes the expression of CX3CR1 in the ceRNA regulatory network. Changes in CX3CR1 expression can affect the process of JEV infection.

We then performed high-throughput RNA-seq of mRNAs in miR-709 transfected BV2 cells (Supplementary Figure S4). In uninfected BV2 cells, 123 genes were downregulated after miR-709 transfection (Figure 4G). In JEV-infected BV2 cells, 12 genes were downregulated after miR-709 transfection include CX3CR1 (Figure 4H). Hence, JEV infection greatly affects the performance of miR-709 on target genes through complex interactions. The downregulated genes in the mock and JEV-infected groups were not completely consistent. The CX3CR1 gene was significantly downregulated only in the JEV-infected group, a finding that was consistent with the results of the ceRNA sequencing analysis of mouse brain tissue. Interestingly, no significant downregulation of CX3CR1 was observed in the mock group. Hence, the downregulation of CX3CR1 was apparently related to JEV infection. We speculate that circRNA-miRNA-mRNA is a very complex regulatory network but not an evenly distributed network; neither the binding of circRNA to miRNAs nor the downstream regulatory target mRNAs are fixed. The regulatory effect of circRNA-miRNA-mRNA may be selective and can only be activated under certain conditions. The Strbp-miR-709-CX3CR1 regulatory pathway in mouse brain may activate by JEV infection.

Through high-throughput sequencing of miR-709-transfected BV2 cells, we further demonstrated the regulatory effect of miR-709 on CX3CR1, a finding that indicates that miR-709-CX3CR1 plays a role in the process of JEV infection in mouse brain. Additionally, as seen from the interaction network for miR-709, there is more than one circRNA that affects miR-709, and CX3CR1 is not the only mRNA targeted by miR-709. The regulatory pathway of X circRNA-miR-709-X mRNA may play different roles in different stages of JEV infection.

**Figure 5:**
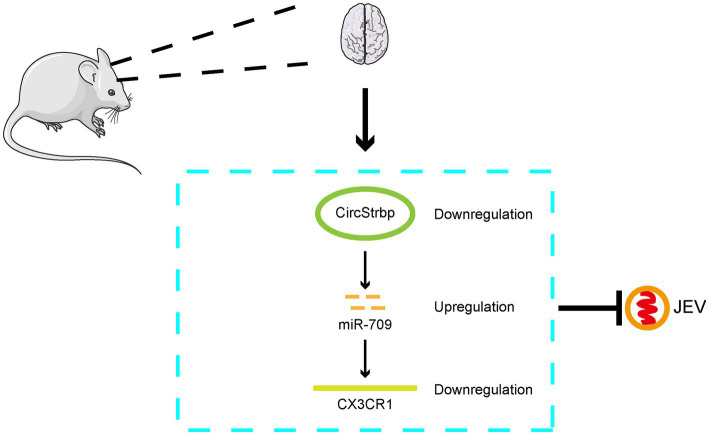
Mode pattern of the effect of CirStrbp-miR-709-CX3CR1 network on the JEV infection in mouse brain.

Finally, we demonstrate that the circStrbp-miR709-CX3CR1 axis changes in response to JEV infection and subsequently influence JEV replication (Figure 5) which is a representative ceRNA net in JEV infection.

## 4 Discussion

Japanese encephalitis is a zoonotic infectious disease that infects humans and animals and is a serious threat in the Asia-Pacific region (Le Flohic et al., 2013). The most important clinical infectious feature of Japanese encephalitis is the occurrence of viral encephalitis. The fine and complex structure of brain tissue has been a challenge of Japanese encephalitis research (Chen et al., 2019). At present, the precise pathogenic mechanism of viral encephalitis caused by JEV infection has not been fully elucidated. In this study, high-throughput sequencing analysis was performed to discover the regulatory role of ceRNAs in the process of JEV infection of brain tissue and to demonstrate the regulatory effect of Strbp-miR-709-CX3CR1 on JEV infection.

High-throughput sequencing was performed using JEV-infected mouse brain tissue, and changes in the expression levels of circRNAs, miRNAs and mRNAs were analyzed. The differentially expressed circRNA, miRNA, and mRNA data obtained herein were not identical to the data reported in previously published studies (Li et al., 2020; Lu et al., 2020; Hu et al., 2021). This difference may be caused by the difference in sampling time of infected mice and the presence of different differentially expressed genes at different stages of JEV infection. The ceRNA regulatory network may also change over time after infection. Li found that the ceRNA pathways of circ_0000220, miR-326-3p and BCL3/MK2/TRIM25 played roles in viral encephalitis, demonstrating that viral encephalitis caused by JEV infection is regulated by a complex ceRNA network (Li et al., 2020). Our study revealed that Strbp-miR-709-CX3CR1 had a regulatory effect on JEV infection. These results demonstrate that the ceRNA network plays different roles during JEV infection.

The abundant circRNAs indicate that ceRNAs are abundant in brain tissue. It is speculated that circRNA-miRNA-mRNA regulation plays an important role in brain tissue. From our experimental results, circRNA-miRNA-mRNA was greatly affected by JEV infection. The high-throughput sequencing results from miR-709 transfected BV2 cells showed that the cellular changes caused by JEV infection affect gene expression and ceRNA network. One possibility of this result is that JEV infection directly affects the production of circRNAs and the expression of miRNAs, resulting in the activation of some specific circRNA-miRNA-mRNA pathways. Another possibility is that JEV infection leads to changes in the expression of some terminal target mRNAs, resulting in the failure to detect the targeting effect of ceRNA network on mRNAs. Based on the results reported herein, the overall expression and differences in expression of miR-709 are more obvious in the early stage of infection, and thus, circRNA-miRNA-mRNA is more likely to play a role in the early stage of virus infection. In the late stage of infection, the regulatory effect of various cytokines and proteins seems to be greater (Bian et al., 2020).

In circRNA-miRNA-mRNA interactions, miRNAs are intermediate molecules. miRNAs can be adsorbed on circRNA sponges and can target mRNAs to generate RNAi(Hansen et al., 2013). We believe that ceRNA analysis centered on miR-709 can explain and validate the circRNA-miRNA-mRNA pathway and its role. Therefore, we transfected miR-709 into BV2 cells and performed high-throughput RNA-seq of mRNAs from transfected cells. The results showed that the changes in CX3CR1 in BV2 cells were the same as the changes in CX3CR1 in mouse brain tissue. Based on the bifunctional chemokine role of CX3CR1, CX3CR1 may play an important role during JEV infection (Liu et al., 2018). Although there is no direct evidence that CX3CR1 is involved in viral inflammation caused by JEV infection, we speculate that changes in CX3CR1 may be associated with inflammation (Doggrell, 2011; Sutti et al., 2019). However, the clear mechanism needs to be further analyzed and verified. By transfecting miR-709 into BV2 cells, we found that miR-709 has a significant anti-JEV effect in BV2 cells. Bioinformatics analysis of the ceRNA network further proved that the antiviral effect of miR-709 is related to CX3CR1. miR-709 also has the potential to target other genes and thus suppress JEV infection (Surendran et al., 2016; Xiong et al., 2022), an issue that requires further experimental verification.

The ceRNA network consisting of circRNA-miRNA-mRNA is very complex, and ceRNAs within the cell generate many potential connections. The biological effects of these ceRNA networks are also complex and multiple. ceRNAs change depending on the organism or cellular state. There are many more ceRNA networks of circStrbp, miR-709, and CX3CR1 were listed in this study (Tables 1^2^), and the changes of molecules related to these networks and the biological effects they produce may have potential roles in JEV infection, which need to be further investigated.

In conclusion, in this study, the circRNA-miRNA-mRNA interaction network in JEV-infected mouse brain tissue and JEV-infected mouse microglia cells was analyzed. The results indicate that the role of circRNA-miRNA-mRNA may change dynamically in different stages of infection and that the circStrbp-miR709-CX3CR1 pathway is associated with the JEV infection process (Figure 5).

## Data availability statement

The authors confirm that the data supporting the findings of this study are available within the article/Supplementary material.

## Ethics statement

Mouse experiments were performed in compliance with the Guidelines on the Human Treatment of Laboratory Animals (Ministry of Science and Technology of the People’s Republic of China, Policy No. 2006 398) and were approved by the Institutional Animal Care and Use Committee at the Shanghai Veterinary Research Institute (IACUC No: Shvri-Pi-0124). The study was conducted in accordance with the local legislation and institutional requirements.

## Author contributions

KL, ZM, and XF: conceived and designed the experiments, writing—review and editing. MC, LK, and TZ: performed the experiments. JZ and DC: data curation. DS, ZL, BL, JW, and YQ: writing—original draft. All authors contributed to the article and approved the submitted version.
