# Supplementary figures and images for: Circular RNA network plays a potential antiviral role in the early stage of JEV infection in mouse brain
Source: Front Microbiol. 2024 Jan 5;14:1165378. doi: 10.3389/fmicb.2023.1165378 (PMC10797004; doi:10.3389/fmicb.2023.1165378)

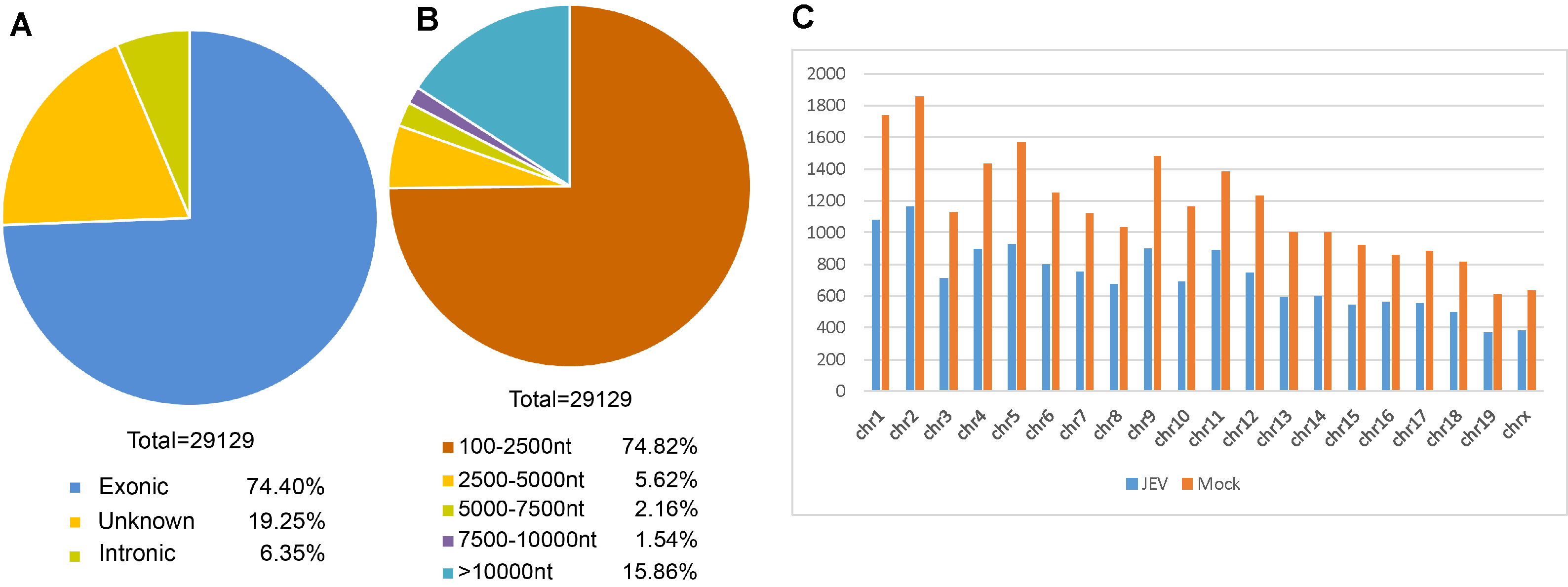

Supplement: Supplementary file 1 [file Image_1.TIF]

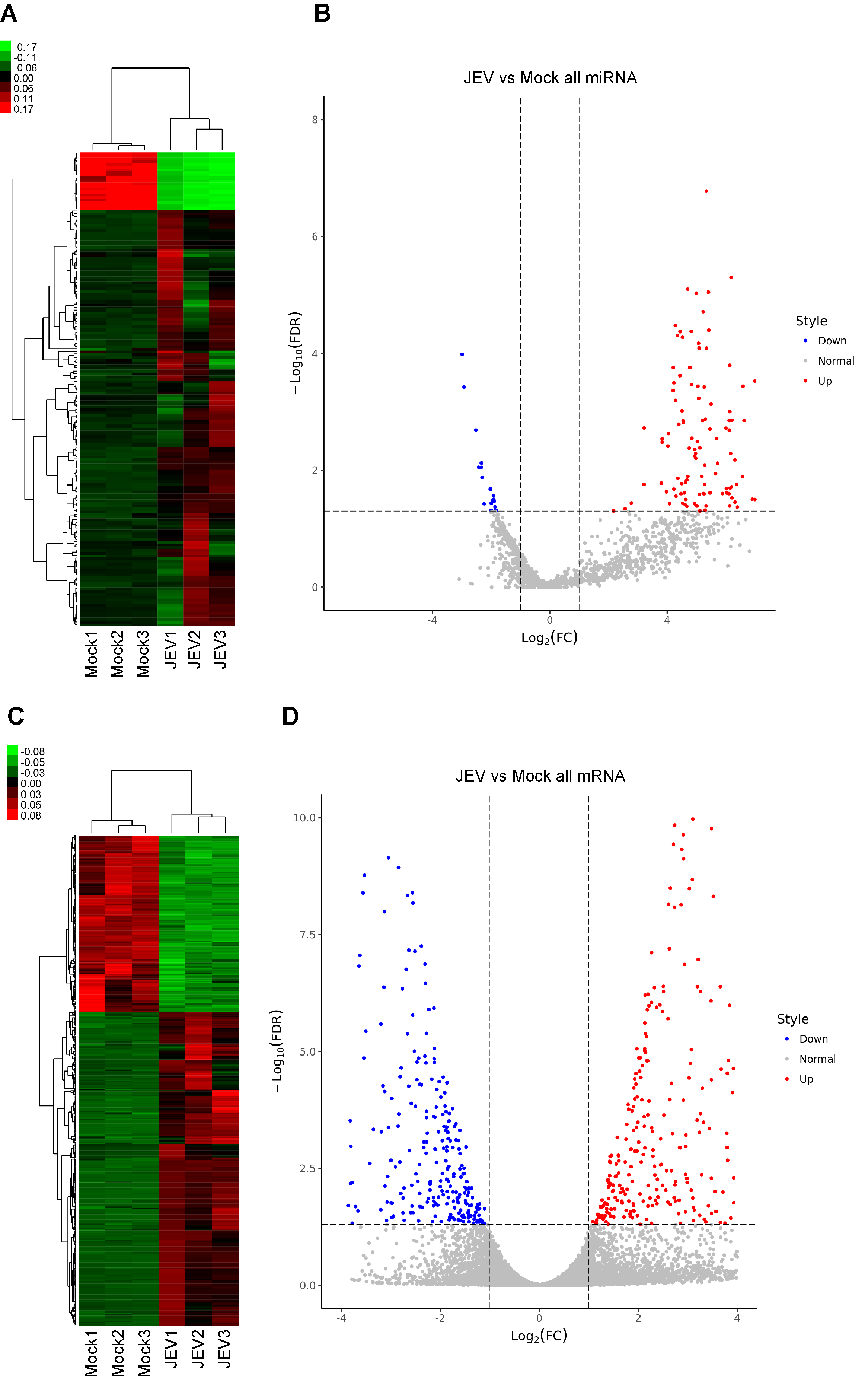

Supplement: Supplementary file 2 [file Image_2.TIF]

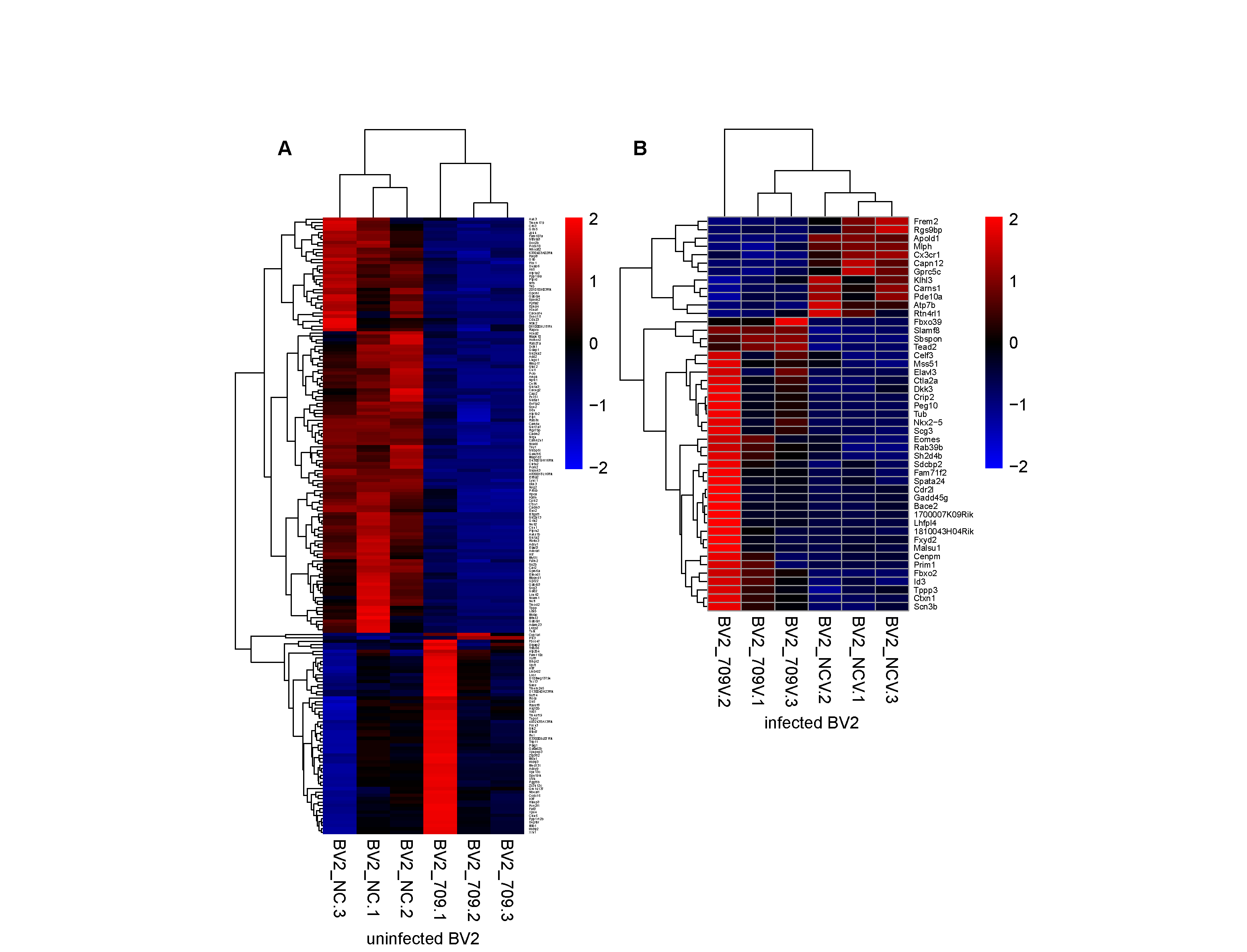

Supplement: Supplementary file 3 [file Image_3.TIF]
